# Measuring and Improving Evidence-Based Patient Care Using a Web-Based Gamified Approach in Primary Care (QualityIQ): Randomized Controlled Trial

**DOI:** 10.2196/31042

**Published:** 2021-12-23

**Authors:** Trever Burgon, Linda Casebeer, Holly Aasen, Czarlota Valdenor, Diana Tamondong-Lachica, Enrico de Belen, David Paculdo, John Peabody

**Affiliations:** 1 QURE Healthcare San Francisco, CA United States; 2 CE Outcomes Birmingham, AL United States; 3 School of Medicine University of California, San Francisco San Francisco, CA United States

**Keywords:** quality improvement, physician engagement, MIPS, case simulation, feedback, value-based care, care standardization, simulation, gamification, medical education, continuing education, outcome, serious game, decision-support

## Abstract

**Background:**

Unwarranted variability in clinical practice is a challenging problem in practice today, leading to poor outcomes for patients and low-value care for providers, payers, and patients.

**Objective:**

In this study, we introduced a novel tool, QualityIQ, and determined the extent to which it helps primary care physicians to align care decisions with the latest best practices included in the Merit-Based Incentive Payment System (MIPS).

**Methods:**

We developed the fully automated QualityIQ patient simulation platform with real-time evidence-based feedback and gamified peer benchmarking. Each case included workup, diagnosis, and management questions with explicit evidence-based scoring criteria. We recruited practicing primary care physicians across the United States into the study via the web and conducted a cross-sectional study of clinical decisions among a national sample of primary care physicians, randomized to continuing medical education (CME) and non-CME study arms. Physicians “cared” for 8 weekly cases that covered typical primary care scenarios. We measured participation rates, changes in quality scores (including MIPS scores), self-reported practice change, and physician satisfaction with the tool. The primary outcomes for this study were evidence-based care scores within each case, adherence to MIPS measures, and variation in clinical decision-making among the primary care providers caring for the same patient.

**Results:**

We found strong, scalable engagement with the tool, with 75% of participants (61 non-CME and 59 CME) completing at least 6 of 8 total cases. We saw significant improvement in evidence-based clinical decisions across multiple conditions, such as diabetes (+8.3%, *P*<.001) and osteoarthritis (+7.6%, *P*=.003) and with MIPS-related quality measures, such as diabetes eye examinations (+22%, *P*<.001), depression screening (+11%, *P*<.001), and asthma medications (+33%, *P*<.001). Although the CME availability did not increase enrollment in the study, participants who were offered CME credits were more likely to complete at least 6 of the 8 cases.

**Conclusions:**

Although CME availability did not prove to be important, the short, clinically detailed case simulations with real-time feedback and gamified peer benchmarking did lead to significant improvements in evidence-based care decisions among all practicing physicians.

**Trial Registration:**

ClinicalTrials.gov NCT03800901; https://clinicaltrials.gov/ct2/show/NCT03800901

## Introduction

Clinical practice variation is recognized as one of the most challenging problems in current practice [1,2]. Unwarranted variability in clinical practice has multiple root causes, starting with the uneven recognition and application of medical knowledge [3,4]. The sheer volume of new research, including nearly 1.4 million papers (or 1 paper every 23 seconds) posted to the National Library of Medicine’s PubMed database in 2019, also makes it virtually impossible for busy practicing physicians to keep their practice up to date [5]. Our own research shows that even after case mix adjustment, practice variation within the same practice is a significant problem, characterized by standard deviations of approximately 10% [6-8]. The good news is that when knowledge and practice gaps are closed, variability declines, adoption of best practices accelerates, and patient outcomes improve [9,10]. Conversely, failing to recognize and address unwarranted variation has deleterious impacts on quality, outcomes, and value [11-16].

The challenge of reducing unwarranted clinical variation has been widely documented across care settings, clinical specialties, Merit-Based Incentive Payment System (MIPS) measures, and geographies. Easy solutions have been tried, including Continuing Medical Education (CME) and maintenance of certification (MOC), performance dashboards, and reminders; however, success has been limited [17]. For example, the most common forms of CME activities, ranging from printed information to didactic presentations and formal conferences, have shown relatively little impact on physician performance [18]. Other engagement strategies, such as multimedia approaches, multiple instructional techniques, repeated exposures, and direct feedback on care decisions, have shown better effectiveness but are difficult to scale and time-intensive for participating physicians [19].

Research using a newer approach—timely feedback on case-based decisions using validated case simulations—has been shown to lead to significant changes in actual practice in randomized controlled trials [6,20,21]. Another research stream has used the motivational aspects of gaming, real-time scoring, digital feedback, leaderboards, and serial competition, which suggests that the gaming approach provides an opportunity to enhance medical education [22-25].

The engagement tool created for this study builds on over 20 years of research using Clinical Performance and Value (CPV) patient simulations [26]. We adapted those lessons to develop a novel web-based patient-simulation platform, known as QualityIQ, which is focused on primary care providers (PCPs) and leverages the serial engagement of case-based learning in CPVs with immediate personalized evidence-based feedback and gamified peer benchmarking. QualityIQ is distinct from the standard gamification approach in that QualityIQ leverages iterative measurement, feedback, and remeasurement over multiple rounds of engagement using the CPV approach. We introduced the QualityIQ tool to PCPs to determine if serial measurement and feedback improved evidence-aligned practice decisions overall and whether it improved specific quality measures included in MIPS. After completing their cases, we determined whether receiving CME credits increased participation in this quality improvement initiative. Finally, we asked the participants directly if they expected to make changes in their actual practice setting after participating in this gamified learning approach.

## Methods

### Study Design

From January through March 2019, we conducted a randomized controlled study of clinical care decisions made by a national sample of PCPs managing typical primary care patients. We asked United States–based, board-certified internal medicine and family medicine physicians to care for four different types of routine primary care cases (diabetes, osteoarthritis [OA], asthma, and musculoskeletal pain). We used the novel web-based QualityIQ patient simulation tool to serially measure provider care decisions for these cases. Physicians were given real-time feedback when they completed their cases to determine the extent to which their care decisions aligned with the latest guidelines. We measured the care decisions judged to be the most critical to high-quality care, namely the workup (laboratory and imaging), diagnosis, and treatment. Gaming elements included a leaderboard for all participants and gift cards for top scores. We took advantage of the prospective design and used a coin flip methodology to randomly assign half of the participants to receive CME and the other half to not receive CME to observe whether this augmented the participation, learning, or standardization effects of serial measurement and feedback.

### Physician Recruitment

From a list of over 10,000 US-based PCPs, we sent out 2000 emails to randomly selected addresses. From this group, we screened potential participants using the following enrollment criteria: (1) is board-certified in internal medicine or family medicine, (2) practices exclusively in primary care, (3) has an active panel of over 1500 patients, and (4) has 2 to 30 years of postresidency experience. In total, 202 providers were eligible, and of these, 141 agreed to participate. The 141 participants were further randomized into 1 of 2 study arms, with 68 in the non-CME control group and 73 in the intervention group that received CME with their participation. Of the 141 physicians who completed the questionnaire and enrolled in the study, 21 began the first week but did not complete their case and were subsequently dropped from the study, leaving 120 enrolled providers who completed at least one week of cases (see Table 1).

**Table 1:** Provider characteristics at baseline (N=120).

Characteristic		Value		*P* value	
		Non-CME^a^ (n=61)	CME (n=59)		
Male, n (%)		43 (70)	38 (64)	.56	
Age >55 years, n (%)		29 (48)	29 (49)	.86	
**Region, n (%)**					.05
	Northeast	20 (33)	16 (27)		
	Midwest	10 (16)	14 (24)		
	West	8 (13)	17 (29)		
	South	23 (38)	12 (20)		
**Locale, n (%)**					.22
	Urban	27 (44)	24 (41)		
	Suburban	26 (44)	32 (54)		
	Rural	8 (13)	3 (5)		
**Specialty, n (%)**					.40
	Family medicine	26 (43)	21 (36)		
	Internal medicine	34 (56)	38 (64)		
	Both	1 (2)	0 (0)		
Attended medical school in the United States, n (%)		49 (80)	42 (71)	.29	
**Practice type, n (%)**					.23
	Solo	15 (25)	10 (17)		
	Group single-specialty	18 (30)	9 (15)		
	Group multispecialty	12 (20)	21 (36)		
	Hospital	5 (8)	7 (12)		
	Academic	6 (10)	6 (10)		
	Other	5 (8)	6 (10)		
Employed by practice, n (%)		42 (69)	51 (87)	.03	
Patients seen/week, mean (SD)		101 (47)	87 (33)	.07	
Receive quality bonus, n (%)		35 (57)	30 (51)	.58	
**Participation in CMS^b^ quality payment programs, n (%)**					
	MIPS^c^	27 (44)	20 (33.9)	.27	
	APM^d^	9 (15)	7 (12)	.79	
	Other	3 (5)	3 (5)	.97	
	None	18 (30)	12 (20)	.29	
Number of rounds of participation, mean (SD)		4.5 (3.2)	6.1 (2.7)	.003	
Participated in ≥6 rounds, n (%)		29 (48)	40 (66)	.045	

^a^CME: Continuing Medical Education.

^b^CMS: Centers for Medicare & Medicaid Services.

^c^MIPS: Merit-Based Incentive Payment System.

^d^APM: Advanced Payment Model.

### QualityIQ Patient Simulation Cases

We created 8 fully automated QualityIQ case simulations and uploaded these cases onto the Qualtrics platform [27]. Each case included evidence-based feedback delivered in real time as physicians progressed through various workup, diagnosis, and treatment decisions. Each case was designed to be completed on a smartphone, tablet, or computer in less than 10 minutes. Each week, all participants cared for the same case.

The 8 cases were developed as pairs of typical cases seen by PCPs in four areas: diabetes, OA, asthma, and pain management (see Table S1 in Multimedia Appendix 1). While each case was unique and required different treatment decisions based on each patient’s presenting symptoms and risk factors, many care decisions were featured in multiple cases (see Table S2 in Multimedia Appendix 2). For example, we included decisions directly related to Medicare 2019 MIPS measures, such as addressing poor hemoglobin A_1c_ control (>9%). We also included general measures that cut across multiple conditions, such as zoster vaccination. By having multiple related scoring items in multiple cases, we were able to track changes over time.

### QualityIQ Scoring and Gamification

The PCPs completed 1 case per week, with weekly email reminders to notify them when the next case opened. Each weekly case consisted of 8 to 10 multiple choice questions covering workup, diagnosis, management, and follow-up decisions, and each question had explicit evidence-based scoring criteria. After each question, physicians received real-time feedback on their care decisions, including the appropriateness of their decision, recommended alternative decisions, and supporting evidence-based references for the preferred care path.

At the end of each week, participants received a detailed score report that included a summary of key evidence-based recommendations for their case, their personal score in the case, and how their care compared to that of their peers. At the start of the study, all participants chose a pseudonym so they could track their scores relative to their peers on a leaderboard that was updated weekly. The top scores in each weekly case were awarded a US $20 electronic gift card from Amazon. The study was completed after the close of the final case.

### Statistical Analysis

The primary outcomes were to measure evidence-based care scores within each case, adherence to MIPS measures, and practice variability among the PCPs caring for the same patient. We were especially keen to determine if the physicians improved their scores on these measures after serial measurements. We also investigated if the availability of CME credit had any effect on participation or performance. Lastly, we asked the participants for their appraisal of the usefulness of the tool in their practice.

For descriptive comparisons between the 2 study arms, we used the chi-square test for significance. To determine significance across cases, we normalized the scores to percentages; a score of 100% indicated that the PCP made all the correct evidence-based decisions without any incorrect decisions, with a possible score of less than 0% if the PCP made more incorrect than correct decisions. We compared these normalized quality-of-care scores across the cases using either multivariate linear regression or the Student *t* test to measure improvements in overall and domain quality of care scores. We also performed an equality of variances test to test for homogeneity of the overall scores. All analyses were conducted in Stata 14.2 (StataCorp LLC).

### Ethics Approval and Consent to Participate

This study was conducted in accordance with ethical standards, approved by the Advarra Institutional Review Board, Columbia, Maryland, and listed on ClinicalTrials.gov (NCT03800901). Informed consent was obtained through electronic signatures from all participants.

## Results

### Physician Demographics

Of the 120 participants in the study, more than two-thirds were male, and 72 (60%) were board certified in internal medicine. Among the demographics and practice characteristics listed in Table 1, we found no significant differences between the two groups except that the CME group had a higher percentage of providers who were employed by their practice (86.4% vs 68.9%; *P*=.03).

All 120 participants cared for one QualityIQ patient in the first week of the project. In the second week, 91 (76%) of the 120 participants completed their second case. After week 2, participation stabilized, with only modest decreases from weeks 3 to 8. Of the 91 participants who completed at least 2 cases, 68 (75%) went on to complete at least 6 of the 8 weekly cases. 58 (48%) physicians completed all 8 cases, and 79 (66%) participated in at least half (n=4) of the cases. When we compared the first week scores between providers who completed 8 weeks of the study to those who only completed the first week of the study, we found no significant difference in their scores (*P*=.37).

The ability to earn CME did not affect recruitment rates. However, once enrolled, those eligible for CME credits completed an average of 1.6 more cases in the project than their non-CME peers (*P*=.003) and were more likely to participate in at least 6 of the weekly rounds (40 of 61, 66.1%, vs 28 of 59, 47.5%; *P*=.045).

In aggregate, female physicians performed significantly better than their male counterparts (+3.1%, *P*=.02), and family medicine diplomates performed better than internal medicine providers (+3.2%, *P*=.008) (see Table 2). We saw no significant difference in overall scores by age, with providers aged over 55 years scoring a nonsignificant 0.7% lower than their younger counterparts (*P*=.56). In our study, those practicing in multispecialty group practices (+6.5%) and those practicing in the Midwest region (+8.1%) scored significantly higher (*P*<.001 for both). PCPs who participated in 6 or more weeks of QualityIQ cases had higher average quality scores than those who participated in 5 or fewer weeks (+5.2%, *P*=.04). However, providers who were randomized into the CME arm of the study did not perform better than those in the non-CME arm (+0.5%, *P*=.84).

**Table 2:** Multivariate linear regression analysis of total QualityIQ scores (as percentages of the maximum score).

Characteristic			Coefficient		*P* value
Male sex			–3.1		.02
Internal medicine physician			–3.2		.008
Age >55 years			–0.7		.56
US-trained physician			–0.1		.97
Midwest region			8.1		<.001
Suburban locale			2.0		.12
Multispecialty group practice			6.5		<.001
Academic practice			4.9		.01
Received quality bonus			­0.8		.50
**Case type^a^**					
	Osteoarthritis	–10.1		<.001	
	Asthma	–6.9		<.001	
	Pain	–8.4		<.001	
Second case of type			6.4		<.001
Participation ≥6 rounds			5.2		.03
CME^b^			0.5		.84
Participation ≥6 rounds * CME			–0.6		.84
Constant			74.7		<.001

^a^Reference case type: diabetes.

^b^CME: Continuing Medical Education.

### Reduction in Variability of Care

Overall, we found a 9.2% reduction in variation between the first and second cases for each case type (*P*=.07). There were different levels of reduction by case type. For example, the relative standard deviation decreased by 37.0% (*P*<.001) in the diabetes cases. When we disaggregated this further, the decreased variation was split fairly evenly between the treatment domain, where the standard deviation decreased by 34.1% (*P*<.001), and a 33.1% reduction was observed in the diagnostic domain (*P*<.001). Variation decreased between the OA and asthma case pairs, but not between the pain cases. In the OA cases, we found a 12.5% relative decrease in variation (*P*=.14), and in the asthma cases, we saw a 15.9% decrease (*P*=.08).

### Quality of Care Improvement Overall and by Case

In the first week of the project, the average score was 77%. When we compared changes in scores among the different case pairs over time (ie, diabetes, OA, asthma, and pain; see Table 3), we found that providers performed 1 to 10 percentage points better in the second case compared to the first. These improvements were statistically significant for patients with diabetes, OA, and asthma but not for the pain case pair. When we looked at the mean scores for the OA and asthma case pairs, we saw a significant increase in the mean scores (Table 3), with the OA case scores improving by 7.6% (*P*=.003) and the asthma scores improving by 10.7% (*P*<.001).

**Table 3:** Summary of QualityIQ results.

Case type and week			Maximum total score		All providers								*P* value	
					n		Mean total score		Percentage of maximum score, mean (SD)					
**Diabetes mellitus**														<.001
	1	350		120		272		77.6 (14.6)						
	7	350		74		301		85.9 (9.2)						
**Osteoarthritis**														.003
	2	270		85		185		68.5 (16.5)						
	4	330		76		251		76.1 (14.6)						
**Asthma**														<.001
	3	260		81		184		70.8 (15.1)						
	6	350		65		285		81.5 (12.7)						
**Pain**														.73
	5	320		72		236		73.7 (12.8)						
	8	310		65		231		74.5 (15.9)						

### Improvement in MIPS-Related Measures

We found that baseline performance on the specific MIPS-related scoring items ranged from 21% for screening and brief counseling for unhealthy alcohol use to 100% for prescribing high blood pressure medication (Table 4). In comparing the two study arms, as well as family medicine versus internal medicine providers, we found no overall differences between the two groups. There were instances of significance, which might be expected with a subanalysis; for example, the CME arm was more than twice as likely (odds ratio [OR] 2.2, 95% CI 1.2-3.8) to order pneumococcal immunization than the non-CME arm, and internal medicine providers were half as likely (OR 0.5, 95% CI 0.3-0.8) to screen for depression.

**Table 4:** Change in Merit-Based Incentive Payment System (MIPS) measures over time.

MIPS measure, category, and name			Ordering, normalized percentage		*P* value
**1. Treatment Diabetes: Hemoglobin A_1c_ (HbA_1c_) Poor Control (>9%)**					<.001
	Week 1	63			
	Week 7	96			
**110. Preventive Care and Screening: Influenza Immunization**					.58
	Week 1	96			
	Week 2	95			
	Week 3	96			
	Week 4	96			
	Week 6	100			
**111. Preventive Care and Screening: Pneumococcal Vaccination Status for Older Adults**					.34
	Week 1	72			
	Week 4	80			
	Week 7	71			
**113. Preventive Care and Screening: Colorectal Cancer Screening**					.72
	Week 1	92			
	Week 4	88			
	Week 7	90			
**117. Treatment: Diabetes: Eye Exam**					<.001
	Week 1	74			
	Week 7	96			
**126. Treatment: Diabetes Mellitus: Diabetic Foot and Ankle Care, Peripheral Neuropathy – Neurological Evaluation**					.07
	Week 1	83			
	Week 7	92			
**134. Preventive Care and Screening: Screening for Depression and Follow-Up Plan**					<.001
	Week 1	84			
	Week 2	71			
	Week 3	70			
	Week 4	96			
	Week 6	83			
	Week 7	96			
	Week 8	95			
**226. Preventive Care and Screening: Tobacco Use: Screening and Cessation Intervention**					.31
	Week 5	93			
	Week 6	97			
**236. Treatment: Controlling High Blood Pressure**					.04
	Week 1	58			
	Week 2	58			
	Week 4	67			
	Week 7	77			
**309. Preventive Care and Screening: Cervical Cancer Screening**					.42
	Week 3	84			
	Week 5	79			
**398. Treatment: Optimal Asthma Control**					.048
	Week 3	99			
	Week 6	98			
**431. Preventive Care and Screening: Unhealthy Alcohol Use: Screening & Brief Counseling**					<.001
	Week 5	21			
	Week 6	50			
	Week 7	53			
	Week 8	55			
**438. Treatment: Statin Therapy for the Prevention and Treatment of Cardiovascular Disease**					.46
	Week 1	92			
	Week 2	78			
	Week 7	95			
**444. Treatment: Medication Management for People with Asthma**					<.001
	Week 3	62			
	Week 6	95			
**474. Preventive Care and Screening: Zoster (Shingles) Vaccination**					<.001
	Week 1	78			
	Week 2	58			
	Week 4	95			
	Week 5	80			
	Week 7	77			

Not surprisingly, measures with baseline performance above 80% showed minimal improvement in subsequent cases. These high-performing measures appeared to be well-established items in primary care practice, such as influenza immunization, colorectal cancer screening, and statin therapy. By contrast, MIPS-related scoring items with baseline performance <80% demonstrated strong and statistically significant improvements through serial measurement and feedback. Notable examples include a 22% increase in diabetic eye examination referrals (*P*<.001), an 11% increase in depression screening (*P*<.001), a 19% increase in appropriate identification of blood pressure goals (*P*=.04), and a 33% increase in evidence-based asthma medication recommendations (*P*<.001). Pneumococcal vaccination for older adults was the lone exception; it started at 72% in the baseline case but did not demonstrate a significant improvement (*P*=.34) in the 3 subsequent cases that included this care decision.

### Physician Survey Results

After the 8 weeks of the project were complete, we asked the physicians about the usefulness of this approach. Of the 120 participants, 62 responded (a 52% response rate). 89% rated the overall quality of the material as good or excellent; 76% reported that they plan to do something in differently in their practice based on what they learned in the cases and the feedback. In addition, participants rated their satisfaction with the gamified weekly leaderboard at 4.1 out of 5.0 on a Likert scale. Importantly, the participants gave the project a net promoter score (NPS) of 59, indicating a strong preference that they would recommend the program to their primary care colleagues.

## Discussion

### Principal Results

Finding effective tools that reduce the variation in clinical practice has been challenging. Traditional CME tools have not shown knowledge retention, and scalable engagement has proven difficult to implement [17,18]. Recent studies have shown that active case-based learning and more interactive techniques, gamification, and deliberate practice show promise in boosting physician engagement, enhancing mastery learning, and improving clinical care quality [23,28-30]. Reducing practice variation and increasing the quality of care patients receive may be most urgently needed in primary care, where the high volume of patients and large breadth of conditions managed are particularly manifest.

This study, which introduced the QualityIQ tool to reduce practice variation, had a few notable findings. Participation rates were high over multiple exposures, with 66% of participants completing at least half of the weekly cases. This is significant because participation was voluntary and offered without any emoluments beyond gamification and recognition on an anonymous leaderboard. The findings also suggest that physicians are interested in efficient and engaging tools that help providers stay abreast of the latest guidelines. Interestingly, the availability of CME and MOC credits had no impact on recruitment into the activity or on performance in the cases, although once a participant joined, they were more likely to complete more cases if they were randomized to CME. We believe that the proliferation of web-based CME opportunities means that fewer physicians need to seek out CME opportunities.

The most significant finding from our study is that iterative measurement, feedback, and remeasurement over multiple rounds of engagement led to significant reductions in care variation (variation reduction by case type: asthma: –15.9%, *P*=.08; osteoarthritis: –12.5%, *P*=.14; diabetes: –37.0%, *P*<.001). There were also broad-based improvements in care decisions from one case to the other (by case type: asthma: +15.1%, *P*<.001; osteoarthritis: +11.1%, *P*=.003; diabetes: +10.7%, *P*<.001). There was no decreased variation or improvement in the pain management cases, which we attribute to two factors: (1) the pain case pairs were too clinically dissimilar (headache and low back pain), and (2) the established clinical guidelines for pain management are less robust that for the other case types. This lack of findings in the pain case type is a strong indicator that the improvements seen in the other case pairs was not simply a “learning effect” bias, wherein participants simply became accustomed to the format.

The specific MIPS-measured care decisions were assessed across multiple cases and also showed improvements with multiple exposures. These improvements extended across preventive and treatment clinical areas, and the measures with the lowest baseline performance showed the strongest improvements. MIPS measures that were adhered to less than 80% of the time at baseline specifically improved between 11% and 33% (*P*<.05). These may be especially important for commonly overlooked items (eg, depression screening) and new items where the guidelines have changed recently (eg, zoster vaccination). Pneumococcal immunization was the outlier, not improving over time from its baseline performance of approximately 70%. This may reflect disagreements with the guideline-based recommendations, which were subsequently updated by the US Centers for Disease Control and Prevention Advisory Committee on Immunization Practices in June 2019, after completion of our data collection [31].

Practice improvement tools only have impact if they are welcome and adopted. Accompanying these improvements, we found corroborating self-reports of practice change among the physician participants and enthusiastic reception of the tool, with an NPS of 59. The NPS is indicative of a user or client’s experience. Users are first asked to rate how likely they are to recommend a service to others. The NPS is then determined by determining the difference between the percentage of promoters (satisfied clients who give a score of 9-10) and the percentage of detractors (dissatisfied clients who gave a score of 0-6). A score above zero can be considered a good score, meaning there are more promoters than detractors, and a score above 50 is considered excellent [32]. In addition, the gamified leaderboard allowing peer-to-peer comparisons using pseudonyms was well received by participants. Another noteworthy finding, given concerns that web-based or digital tools may not reach older physicians, is that physicians over the age of 55 years performed as well as other providers, suggesting that the approach may be broadly applicable to practicing PCPs at various stages of their career.

### Limitations

There are limitations to this validation study that are worth noting. Although an impressive 76% of participants reported making changes to their practice based on their participation in the QualityIQ cases and feedback, the study was not designed to interrogate practice or patient-level records to validate these improvements. This important work will be left to future studies. In addition, this 8-week curriculum covered a number of cases typically seen in primary care, but it did not include an exhaustive range of high-priority topics. This could be addressed through longer-term studies, potentially in partnership with health systems or physician groups. The project was designed to simulate actual practice decisions through simulations rather than create a fully validated examination. As such, questions formulated around areas of clinical relevance were tied to typical practice patterns. Psychometric validation of the questions was not performed but could be a priority for future academic research applications of the tool. In addition, although PCPs play a critically important role in quality improvement, there are significant opportunities to improve care quality among specialist physicians, medical trainees, nurses, and other health care professionals. Future work will elucidate the impact of this engagement model in these other settings.

### Conclusions

In recognition of the vital role of primary care, multiple programs from government and nongovernment agencies have prioritized primary care practice improvement as essential to care transformation efforts to improve care quality and value. In this study, we have shown that short case simulations delivering real-time personalized feedback and gamified peer benchmarking are very well received by practicing primary care physicians and lead to significant improvements in evidence-based care decisions. Importantly, as the QualityIQ scores increased, the unwarranted variation between providers decreased, which is a “holy grail” in efforts to build high-quality, high-reliability primary care networks. As a web-based, scalable engagement tool, this model may be of interest to health systems, payers, policy makers, patient advocacy groups, and life science companies looking to collaborate with providers in practice change efforts to improve the quality, value, and consistency of care.

### Data Availability

The data sets used to support the findings of this study are available from the corresponding author upon reasonable request.

## Appendix

Multimedia Appendix 1 QualityIQ case summaries.

Multimedia Appendix 2 Merit-Based Incentive Payment System measures by case.

Multimedia Appendix 3 CONSORT-eHEALTH checklist (V 1.6.1).

## Abbreviations

CME: continuing medical education
CPV: Clinical Performance and Value
MIPS: Merit-Based Incentive Payment System
MOC: maintenance of certification
NPS: net promoter score
OA: osteoarthritis
OR: odds ratio
PCP: primary care provider
