# Supplementary material for: Measuring and Improving Evidence-Based Patient Care Using a Web-Based Gamified Approach in Primary Care (QualityIQ): Randomized Controlled Trial
Source: J Med Internet Res. 2021 Dec 23;23(12):e31042. doi: 10.2196/31042 (PMC8738991; doi:10.2196/31042)
Supplement: Multimedia Appendix 1 [file jmir_v23i12e31042_app1.docx]

**Table S1. QualityIQ Case Summaries.**

**Diabetes**

*Case 1A*

58-year-old man with hypertension and poorly-controlled diabetes resulting in diabetic kidney disease and diabetic neuropathy. Core treatment elements include guideline-based agents for optimal blood sugar control, RAAS blockade, cardiovascular risk reduction, and diabetes-related non-pharmacologic interventions.

*Case 1B*

68-year-old man with hypertension, CKD, HF, and poorly-controlled diabetes. Key treatment elements include shifting to insulin therapy, starting beta blockers for newly-diagnosed HF, cardiovascular risk reduction, diabetes education, nephrology referral, and screening for microvascular complications.

**Hypertension**

*Case 2A*

69-year-old woman with hypertension who experiences orthostatic dizziness related to thiazide intake. Key treatment elements include guideline-based medication titration for blood pressure control, cardiovascular risk reduction, fall risk assessment and intervention, and preventive care (including immunizations and age-appropriate disease screening).

*Case 2B*

54-year-old man with blood pressure elevation aggravated by NSAID use for knee osteoarthritis and decongestant intake for sinusitis. Core treatment strategies include withdrawal of NSAIDs and decongestants, addition of another first-line antihypertensive towards a guideline-based target, as well as intranasal steroids and nasal irrigation for his acute sinusitis.

**Asthma**

*Case 3A*

29-year-old woman with known asthma who presents with a mild exacerbation requiring an increase in the dose of maintenance inhaled steroid/LABA and an increase in the frequency of reliever SABA. Other key treatment elements include avoidance of inappropriate imaging and antibiotic use for sinusitis, emphasis on non-pharmacologic interventions such as asthma education, and preventive care (including immunizations).

*Case 3B*

28-year-old man with obesity and asthma who presents with a mild asthma exacerbation and reflux symptoms. Core therapeutic strategies include guideline-based asthma medication titration, proton pump inhibitor treatment, and non-pharmacologic intervention such as weight reduction, regular aerobic physical activity, and smoking cessation. Preventive care (including immunizations) and appropriate screening for at-risk conditions are also highlighted.

**Acute Pain**

*Case 4A*

55-year-old woman with severe pulsating headaches who should be diagnosed with migraine headache, based on clinical grounds (imaging not indicated). Recommended treatment includes migraine-specific medications, avoidance of opioids, and migraine prophylaxis. Preventative care (including immunizations and age-appropriate screening) is also highlighted.

*Case 4B*

38-year-old man on HAART who presents with acute low back pain. Key management elements include avoidance of unnecessary imaging and guideline-based non-pharmacologic methods, complementary pharmacologic treatment, and avoidance of opioids. Preventative care (including age- and disease-appropriate screening) is also emphasized.

**Abbreviations:**

CKD: Chronic kidney disease

HAART: Highly active antiretroviral therapy

HF: Heart failure

LABA: Long-acting beta-agonist

NSAID: Non-steroidal anti-inflammatory drug

RAAS: Renin-angiotensin-aldosterone system

SABA: Short-acting beta-agonist
