# Supplementary material for: Measuring and Improving Evidence-Based Patient Care Using a Web-Based Gamified Approach in Primary Care (QualityIQ): Randomized Controlled Trial
Source: J Med Internet Res. 2021 Dec 23;23(12):e31042. doi: 10.2196/31042 (PMC8738991; doi:10.2196/31042)
Supplement: Multimedia Appendix 2 [file jmir_v23i12e31042_app2.docx]

**Table S2. MIPS Measures by Case**

| Case Type | Number of MIPS measures | MIPS Measures |
| --- | --- | --- |
| DM | 9 | Diabetes: Hemoglobin A1c (HbA1c) Poor Control (>9%)  Diabetes: Eye Exam  Diabetes Mellitus: Diabetic Foot and Ankle Care, Peripheral Neuropathy – Neurological Evaluation  Controlling High Blood Pressure  Statin Therapy for the Prevention and Treatment of Cardiovascular Disease  Preventive Care and Screening: Screening for Depression and Follow-Up Plan  Preventive Care and Screening: Influenza Immunization  Pneumococcal Vaccination Status for Older Adults  Colorectal Cancer Screening  Zoster (Shingles) Vaccination |
|  | 10 | Diabetes: Hemoglobin A1c (HbA1c) Poor Control (>9%)  Diabetes: Eye Exam  Diabetes Mellitus: Diabetic Foot and Ankle Care, Peripheral Neuropathy – Neurological Evaluation  Controlling High Blood Pressure  Statin Therapy for the Prevention and Treatment of Cardiovascular Disease  Pneumococcal Vaccination Status for Older Adults  Colorectal Cancer Screening  Preventive Care and Screening: Screening for Depression and Follow-Up Plan  Preventive Care and Screening: Unhealthy Alcohol Use: Screening & Brief Counseling  Zoster (Shingles) Vaccination |
| OA | 8 | Controlling High Blood Pressure  Osteoarthritis: Function and pain assessment  Adult sinusitis: antibiotic prescribed for acute sinusitis (overuse)  Adult sinusitis: CT for acute sinusitis (overuse)  Statin Therapy for the Prevention and Treatment of Cardiovascular Disease  Preventive Care and Screening: Influenza Immunization  Preventive Care and Screening: Screening for Depression and Follow-Up Plan  Zoster (Shingles) Vaccination |
|  | 10 | Controlling High Blood Pressure  Osteoarthritis: Function and pain assessment  Adult kidney disease: blood pressure management  Falls: Risk assessment  Falls: Plan of care  Preventive Care and Screening: Influenza Immunization  Pneumococcal Vaccination Status for Older Adults  Colorectal Cancer Screening  Preventive Care and Screening: Screening for Depression and Follow-Up Plan  Zoster (Shingles) Vaccination |
| Asthma | 7 | Optimal Asthma Control  Medication Management for People with Asthma  Adult sinusitis: antibiotic prescribed for acute sinusitis (overuse)  Adult sinusitis: CT for acute sinusitis (overuse)  Preventive Care and Screening: Influenza Immunization  Preventive Care and Screening: Screening for Depression and Follow-Up Plan  Cervical Cancer Screening |
|  | 9 | Optimal Asthma Control  Medication Management for People with Asthma  Screening for high blood pressure and follow-up  Body mass index screening and follow-up plan  HIV/AIDS: Sexually Transmitted Disease Screening for Chlamydia, Gonorrhea, and Syphilis  Preventive Care and Screening: Influenza Immunization  Preventive Care and Screening: Screening for Depression and Follow-Up Plan  Preventive Care and Screening: Tobacco Use: Screening and Cessation Intervention  Preventive Care and Screening: Unhealthy Alcohol Use: Screening & Brief Counseling |
| Pain | 10 | Overuse of neuroimaging for patients with primary headache and normal neurologic exam  Evaluation or interview for risk of opioid misuse  Pain brought under control within 48 hours  Controlling High Blood Pressure  Preventive Care and Screening: Influenza Immunization  Preventive Care and Screening: Screening for Depression and Follow-Up Plan  Preventive Care and Screening: Tobacco Use: Screening and Cessation Intervention  Cervical Cancer Screening  Preventive Care and Screening: Unhealthy Alcohol Use: Screening & Brief Counseling  Zoster (Shingles) Vaccination |
|  | 7 | Use of Imaging for Low Back Pain  Pain Assessment and Follow-Up  Depression remission at 12 months  HIV/AIDS: Sexually Transmitted Disease Screening for Chlamydia, Gonorrhea, and Syphilis  Preventive Care and Screening: Screening for Depression and Follow-Up Plan  Preventive Care and Screening: Unhealthy Alcohol Use: Screening & Brief Counseling  Preventive Care and Screening: Unhealthy Alcohol Use: Screening & Brief Counseling |
